# Risk factors and prediction of hypoglycaemia using the Hypo-RESOLVE cohort: a secondary analysis of pooled data from insulin clinical trials

**DOI:** 10.1007/s00125-024-06177-6

**Published:** 2024-05-25

**Authors:** Joseph Mellor, Dmitry Kuznetsov, Simon Heller, Mari-Anne Gall, Myriam Rosilio, Stephanie A. Amiel, Mark Ibberson, Stuart McGurnaghan, Luke Blackbourn, William Berthon, Adel Salem, Yongming Qu, Rory J. McCrimmon, Bastiaan E. de Galan, Ulrik Pedersen-Bjergaard, Joanna Leaviss, Paul M. McKeigue, Helen M. Colhoun

**Affiliations:** 1 Usher Institute, College of Medicine and Veterinary Medicine, University of Edinburgh, Edinburgh, UK; 2 Swiss Institute of Bioinformatics, Lausanne, Switzerland; 3 Division of Clinical Medicine, University of Sheffield, Sheffield, UK; 4 Medical & Science, Insulin, Clinical Drug Development, Novo Nordisk A/S, Soeberg, Denmark; 5 Eli Lilly and Company, Diabetes Medical Unit, Neuilly sur seine, France; 6 Department of Diabetes, School of Cardiovascular and Metabolic Medicine and Sciences, Faculty of Life Sciences and Medicine, King’s College London, London, UK; 7 Institute of Genetics and Cancer, College of Medicine and Veterinary Medicine, University of Edinburgh, Edinburgh, UK; 8 RW Data Assets, AI & Analytics (AIA), Novo Nordisk A/S, Soeberg, Denmark; 9 Eli Lilly and Company, Indianapolis, IN USA; 10 Systems Medicine, School of Medicine, University of Dundee, Dundee, UK; 11 Department of Internal Medicine, Division of Endocrinology and Metabolic Disease, Maastricht University Medical Center, Maastricht, the Netherlands; 12 Institute of Clinical Medicine, University of Copenhagen, Copenhagen, Denmark; 13 School of Health and Related Research (ScHARR), University of Sheffield, Sheffield, UK

**Keywords:** Hypoglycaemia, Hypo-RESOLVE, Prediction modelling

## Abstract

**Aims/hypothesis:**

The objective of the Hypoglycaemia REdefining SOLutions for better liVES (Hypo-RESOLVE) project is to use a dataset of pooled clinical trials across pharmaceutical and device companies in people with type 1 or type 2 diabetes to examine factors associated with incident hypoglycaemia events and to quantify the prediction of these events.

**Methods:**

Data from 90 trials with 46,254 participants were pooled. Analyses were done for type 1 and type 2 diabetes separately. Poisson mixed models, adjusted for age, sex, diabetes duration and trial identifier were fitted to assess the association of clinical variables with hypoglycaemia event counts. Tree-based gradient-boosting algorithms (XGBoost) were fitted using training data and their predictive performance in terms of area under the receiver operating characteristic curve (AUC) evaluated on test data. Baseline models including age, sex and diabetes duration were compared with models that further included a score of hypoglycaemia in the first 6 weeks from study entry, and full models that included further clinical variables. The relative predictive importance of each covariate was assessed using XGBoost’s importance procedure. Prediction across the entire trial duration for each trial (mean of 34.8 weeks for type 1 diabetes and 25.3 weeks for type 2 diabetes) was assessed.

**Results:**

For both type 1 and type 2 diabetes, variables associated with more frequent hypoglycaemia included female sex, white ethnicity, longer diabetes duration, treatment with human as opposed to analogue-only insulin, higher glucose variability, higher score for hypoglycaemia across the 6 week baseline period, lower BP, lower lipid levels and treatment with psychoactive drugs. Prediction of any hypoglycaemia event of any severity was greater than prediction of hypoglycaemia requiring assistance (level 3 hypoglycaemia), for which events were sparser. For prediction of level 1 or worse hypoglycaemia during the whole follow-up period, the AUC was 0.835 (95% CI 0.826, 0.844) in type 1 diabetes and 0.840 (95% CI 0.831, 0.848) in type 2 diabetes. For level 3 hypoglycaemia, the AUC was lower at 0.689 (95% CI 0.667, 0.712) for type 1 diabetes and 0.705 (95% CI 0.662, 0.748) for type 2 diabetes. Compared with the baseline models, almost all the improvement in prediction could be captured by the individual’s hypoglycaemia history, glucose variability and blood glucose over a 6 week baseline period.

**Conclusions/interpretation:**

Although hypoglycaemia rates show large variation according to sociodemographic and clinical characteristics and treatment history, looking at a 6 week period of hypoglycaemia events and glucose measurements predicts future hypoglycaemia risk.

**Graphical Abstract:**

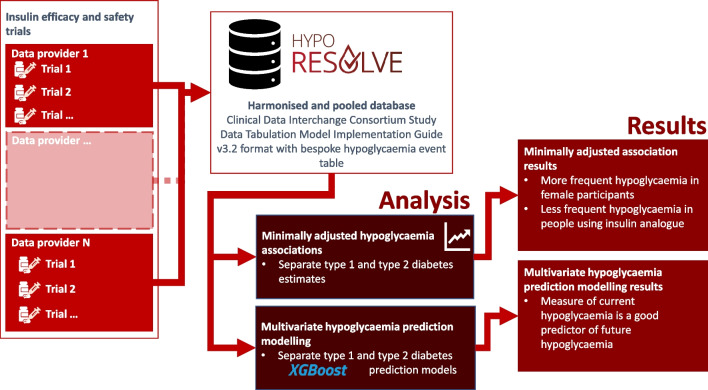

**Supplementary Information:**

The online version contains peer-reviewed but unedited supplementary material available at 10.1007/s00125-024-06177-6.


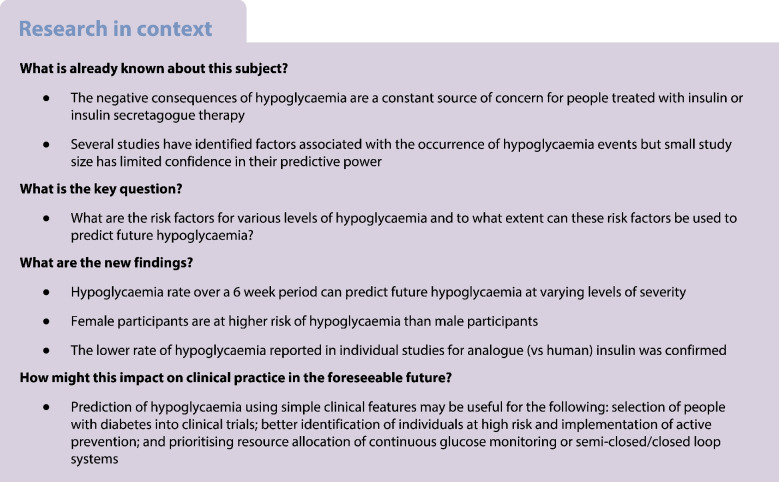


## Introduction

Hypoglycaemia is an acute complication of diabetes management that may occur as a consequence of insulin or insulin secretagogue therapy [1]. The negative consequences (physical, psychological and behavioural) of hypoglycaemia are a constant source of concern for people treated with these agents and those who provide care for them [2]. For some individuals, fear of hypoglycaemia prevents them from achieving recommended glycaemic targets, increasing their risk of a range of complications [3]. Conversely, fear of hyperglycaemia can lead to over-use of insulin and more frequent hypoglycaemia [4]. A greater understanding of predictors of hypoglycaemia may enable healthcare professionals to better advise people with diabetes how to avoid hypoglycaemia events while also maintaining optimal glycaemic control. Prediction of hypoglycaemia risk could also inform clinical decision making and clinical trial entry criteria or enable hypoglycaemia prevention strategies. Several studies [5–7] have identified factors associated with the occurrence of hypoglycaemia events but further understanding of causal relationships between clinical risk factors and, in particular, a range of definitions for hypoglycaemia outcomes, would enable targeted interventions for individuals at increased hypoglycaemia risk.

Hypoglycaemia rates should be, and often are, used as a safety endpoint in clinical trials investigating the effect of glucose-lowering therapies. Accurate identification of the efficacy of different insulins in not only maintaining glycaemic control but also limiting hypoglycaemia is of fundamental importance to people with type 1 diabetes or insulin-treated type 2 diabetes. Consequently, a thorough understanding of hypoglycaemia risk and its predictors across clinical trial participants at baseline could be of interest to evaluate hypoglycaemia events during the trial.

The EU-funded Hypoglycaemia REdefining SOLutions for better liVEs (Hypo-RESOLVE) project brought people with diabetes together with academic, clinical and industry partners with the joint goal of identifying and quantifying predictors and consequences of hypoglycaemia [1]. As part of this initiative a database was created of clinical trial data provided by several pharmaceutical and medical device industry partners, involving people with type 1 or type 2 diabetes in whom data on hypoglycaemia events had been captured during the trial. In this pre-specified analysis, we leveraged this pooled dataset to examine the association of clinical variables collected at study baseline and during the trial with incident hypoglycaemia events and quantified how well the events could be predicted with these data.

## Methods

### Data and cohort

Trial data from 26 clinical trials involving 12,247 people living with type 1 diabetes and 65 trials involving 34,007 people living with type 2 diabetes were provided by industry partners. All trials involved people with diabetes who were taking glucose-lowering medication with hypoglycaemia risk, mostly insulin. The raw trial data were standardised, harmonised and pooled in a unique hypo-RESOLVE database by the Swiss Institute of Bioinformatics, using the Clinical Data Interchange Consortium (CDISC) Study Data Tabulation Model Implementation Guide (SDTMIG 3.2) format [8] (see electronic supplementary material [ESM] Methods for details). In addition, the bespoke domain XH was created for hypoglycaemia event data, obtained from self-recorded episodes in participants’ diaries and serious adverse event declaration from clinical trials. The trials did not use continuous glucose monitoring (CGM). Some episodes were asymptomatic episodes noted on self-monitored blood glucose that met the agreed thresholds for hypoglycaemia, and some were symptomatic episodes. Level 3 (see below) episodes did not require a blood glucose measurement as this was not part of the definition, although it was often recorded. Some level 3 episodes were derived also from serious adverse event reporting. Each hypoglycaemia event was characterised by an event date, a blood glucose measurement (if available) and self-treatment status.

Despite the availability of raw data from each clinical trial, many trials had idiosyncratic data structures or collection procedures that precluded data harmonisation into the pooled database. These issues resulted in the exclusion of certain individuals and covariates due to the high levels of missingness introduced when integrating the data from these trials. We therefore first excluded individuals who met the following criteria: did not pass trial screening; lacked observation start or end dates; had missing age, sex or diabetes duration information; or had more than 20% missingness for hypoglycaemia event data. A hypoglycaemia event was considered missing if the event lacked a date of occurrence or it lacked a glucose measurement while simultaneously being either denoted as a self-treated event or the self-treatment status was missing.

### Definitions of hypoglycaemia

Blood glucose measurements and whether assistance was required to handle each hypoglycaemia event was used to define hypoglycaemia in our analyses, irrespective of each trial’s own definition in the pooled dataset.

The International Hypoglycaemia Study Group (IHSG) [9] proposed three levels of hypoglycaemia that have been accepted recently by the European Medicines Agency (EMA) [10] and, as draft guidance, by the US Food and Drug Administration (FDA) [11]. Currently, these are as follows:

- Level 1 hypoglycaemia alert events, defined as any event with a recorded blood glucose level of less than 3.9 mmol/l but not less than 3.0 mmol/l
- Level 2 hypoglycaemia events, defined as any hypoglycaemia event with a recorded blood glucose level below 3.0 mmol/l
- Level 3 hypoglycaemia events (severe hypoglycaemia), defined as any hypoglycaemia event in which the individual was unable to self-treat due to severe cognitive impairment, irrespective of glucose measurement

Within the pooled clinical trial dataset, level 3 was any event in the XH table that was both symptomatic and not self-treated.

Using these levels, we considered three separate classifications of hypoglycaemia event in our analyses:

- Level 1 or worse: any hypoglycaemia event meeting the criteria of either level 1, level 2 or level 3
- Level 2 or worse: any hypoglycaemia event meeting the criteria of either level 2 or level 3
- Level 3

### Candidate covariates

We sought to examine the association of subsequent hypoglycaemia with a wide range of variables that have either been previously reported as associated with hypoglycaemia or for which an association might reasonably be expected and for which data were available in a sufficient number of trials or participants. In addition to age, sex (as reported by the investigator of the clinical trial) and diabetes duration we considered the following candidate covariates in our analysis: total daily insulin dose; insulin regimen (basal, basal bolus, or premix); insulin origin (human vs analogue); self-monitored blood glucose; variability based on self-monitored blood glucose; HbA_1c_; eGFR as defined by the CKD-EPI equation [12]; systolic BP; diastolic BP; medical history of complications of diabetes (CVD, retinopathy, neuropathy, nephropathy); total cholesterol; LDL-cholesterol; HDL-cholesterol; triglycerides; BMI; ethnicity; and use of concomitant medications (glucose-lowering drugs, antihypertensives, systemic antibiotics, systemic oral anti-inflammatory agents, psychoactive agents, sex hormones, anti-epilepsy drugs, antithyroid drugs, cessation of systemic steroids).

Medical history covariates were defined by relevant Medical Dictionary for Regulatory Activities (MedDRA) terms and drug categories were defined using ATC codes (ESM Tables 1, 2).

Since we considered that an individual’s recent history of hypoglycaemia was likely to be an important predictor of future hypoglycaemia events, and since this information would ordinarily be available in a clinical setting, we used the first 6 weeks following the date of randomisation into their clinical trials (an arbitrary minimum time period in which to estimate a typical hypoglycaemia baseline) to obtain measures of baseline hypoglycaemia incidence, baseline blood glucose and blood glucose variability for each participant. Follow-up time and events after this first 6 weeks were then used in the evaluation of associations and predictions. A simple hypoglycaemia score was arbitrarily defined as the weighted sum of the number of level 1, 2 and 3 hypoglycaemia event counts in a 6 week period, with a 1:2:3 ratio between level 1, 2 and 3 event counts, respectively. Since the hypoglycaemia score was estimated after randomisation, the independent effect of the randomised insulin origin and regimen was not distinguishable in multivariate models.

Blood glucose variability was characterised by the CV calculated as the ratio of the SD to the mean of blood glucose within a 6 week time interval, as the CV is one of the most commonly used measures of this variable.

### Missingness, evaluability and imputation

All continuous covariates were categorised as either having an evaluable continuous value or as being missing. For covariates such as sex and ethnicity, the covariate was either considered evaluable or missing. For drug exposure and medical history covariates, if at least one person in a given trial had the covariate recorded we considered all the participants in that trial to be evaluable for these covariates, otherwise we regarded the covariates as non-evaluated in a given trial.

Covariates were imputed on a per-trial basis using the R package Amelia (version 1.7.6; https://cran.r-project.org/web/packages/Amelia/index.html), provided the covariate was present for at least 80% of participants in that trial.

### Statistical methods

#### Data set-up

We structured our data in a longitudinal format, with time slices of 6 weeks. Time was measured relative to the entry date of each individual. Individuals exited the study at the earliest of the end of participation in the clinical trial or date of death.

#### Rates of hypoglycaemia

We first examined how much heterogeneity there was in the crude incidence rates of hypoglycaemia events at the three levels across clinical trials. A large degree of heterogeneity was expected given the varying entry criteria across trials and this had important implications for the potential of confounding of association by trial number.

#### Minimally adjusted associations with hypoglycaemia

To quantify the association of a range of clinical covariates with each hypoglycaemia outcome, we used multivariate generalised linear mixed models (GLMMs). For each analysis, the number of hypoglycaemia events experienced by an individual during a time slice was the measured outcome. We employed a Poisson mixed model for our analysis with random intercept for individual to account for any over-dispersion since the count of hypoglycaemia events is time-updated. This is as opposed to negative-binomial regression, which erroneously assumes that observations across individuals are exchangeable.

All analyses were performed for type 1 and type 2 diabetes separately. Separate GLMMs, adjusted for age, sex and diabetes duration, for participants with known insulin regimen were fit to investigate the adjusted association of each candidate covariate after imputation. We adjusted models for study identifier to account for confounding due to different trial entry criteria and populations. The covariate value for the first 6 weeks from study entry was used in the models, with only events after this time being considered in the analysis. The hypoglycaemia event rate was assumed to be constant across time slices for the same participant.

#### Prediction modelling

For multivariate prediction modelling, further exclusion criteria to the cohort were applied for each analysis separately. We dropped the following from consideration in our analysis: participants with unknown insulin regimen; any covariates with more than 20% missingness; any individual who had missingness in any retained candidate covariate; and concomitant medications where less than 5% of individuals were recorded as using them. We also dropped all participants in studies where there were 15 or fewer hypoglycaemia events in total across the study of the level corresponding to the outcome of the specific analysis, as such trials had too little information to contribute to the model. Data were partitioned in a 70:30 training:test split stratified by trial.

The prediction task was to predict the number of hypoglycaemia events from start of study (6 weeks post-randomisation) to end of study.

For each hypoglycaemia outcome, we fitted three models: (1) a baseline model that included age, sex, diabetes duration and study identifier; (2) a baseline model also including the hypoglycaemia score; and (3) a full model (also including the hypoglycaemia score). For the full model, all covariates meeting missingness criteria, separately for type 1 and type 2 diabetes, were included from the candidate set. In all models, the participant was included as a random effect.

Although our models predicted the number of hypoglycaemia events, for summarisation purposes the AUC for the binary outcome of the number of hypoglycaemia events at the threshold of being more or less than the 90th centile within the trial was computed. Prediction modelling included 18 models (two diabetes cohorts, three prediction outcomes and three comparator model types).

XGBoost implements a tree-based gradient boosting algorithm to fit predictive models [13]. We fitted XGBoost models using the training split to perform a threefold cross-validation grid-search (parameters are given in ESM Methods). The selected model was then evaluated on the test split where test log(likelihood) and AUC were evaluated. The difference in test log(likelihood) between two models provided the strength of evidence that one model had greater predictive performance than the other; a difference in test log(likelihood) of 6.9 natural log units is asymptotically equivalent to a *p* value less than 0.005 for comparison of nested models [14].

## Results

### Data availability

The data, after exclusion criteria, consisted of 46,254 individuals with 31,577 person-years observed (mean follow-up of 0.68 years), with data collected from 90 unique trials. There were 12,247 participants with type 1 diabetes from 26 clinical trials and 34,007 participants with type 2 diabetes from 65 trials. During follow-up, there was a total of 841,401 (approximately 88 per person-year) and 309,655 (approximately 14 per person-year) level 1 or worse hypoglycaemia events in the type 1 and type 2 diabetes cohorts, respectively. For level 2 or worse, there were 334,086 (approximately 35 per person-year) and 76,987 (approximately three per person-year) hypoglycaemia events in the type 1 and type 2 diabetes cohorts, respectively. For level 3 hypoglycaemia, there were 4719 (approximately 0.49 per person-year) and 3414 (approximately 0.15 per person-year) events in the type 1 and type 2 diabetes cohorts, respectively.

Cohort characteristics are presented in Tables 1 and 2, separated by type of diabetes, and provide numbers of evaluable participants after imputation for each covariate.

**Table 1:** Cohort characteristics for individuals with type 1 diabetes where reported values are calculated from the first 6 weeks since trial entry

Covariate	Median (IQR)/*N* (%)	Evaluable participants	Evaluable studies
Age, years	37 (25)	12,247	26
Female sex	5451 (44.51)	12,247	26
Ethnicity, non-White	1594 (13.96)	11,416	23
Diabetes duration, years	13.3 (17.2)	12,247	26
HbA_1c_, mmol/mol	61.75 (14.43)	11,710	25
HbA_1c_, %	7.8 (1.32)	11,710	25
Blood glucose, mmol/l	8.73 (2.34)	10,618	22
eGFR, ml/min per 1.73 m^2^	103.56 (31.49)	10,507	21
Systolic BP, mmHg	120.67 (19.5)	11,649	24
Diastolic BP, mmHg	74 (12)	11,649	24
BMI, kg/m^2^	24.93 (6.17)	11,649	24
Total cholesterol, mmol/l	4.63 (1.2)	8809	16
LDL-cholesterol, mmol/l	2.57 (1.03)	8809	16
HDL-cholesterol, mmol/l	1.58 (0.59)	8809	16
Triglycerides, mmol/l	0.88 (0.58)	7613	14
Total insulin dose, U/day	45 (36.36)	11,385	23
Insulin origin			
Human	249 (2.25)	11,059	24
Analogue	10,041 (90.79)	11,059	24
Human analogue	769 (6.95)	11,059	24
Insulin regimen: basal bolus	11,059 (100)	11,059	24
Concomitant disease at baseline			
CVD	3338 (28.3)	11,796	25
Retinopathy	2605 (22.78)	11,433	24
Neuropathy	2160 (20.67)	10,450	22
Nephropathy	1471 (14.8)	9940	20
Concomitant medication			
Psychoactive drug	1137 (9.86)	11,532	24
Glucose-lowering drug	36 (0.41)	8777	15
Anti-epileptic drug	215 (1.95)	11,041	22
Anti-inflammatory drug	2457 (21.31)	11,532	24
Antithyroid drug	49 (0.49)	9935	18
Antihypertensive drug	3117 (27.03)	11,532	24
Antibiotic	66 (0.57)	11,532	24
Sex hormone	570 (4.94)	11,532	24

Median and IQR are reported for continuous variables; IQR is given as the distance between the 25th and 75th percentile; frequency and percentage are reported for categorical variables

**Table 2:** Cohort characteristics for individuals with type 2 diabetes where reported values are calculated from the first 6 weeks since trial entry

Covariate	Median (IQR)/*N* (%)	Evaluable participants	Evaluable studies
Age, years	59 (14)	34,007	65
Female sex	15,942 (46.88)	34,007	65
Ethnicity: non-White	9840 (32.88)	29,927	54
Diabetes duration, years	10.7 (9.57)	34,007	65
HbA_1c_, mmol/mol	64.49 (14.21)	29,756	63
HbA_1c_, %	8.05 (1.3)	29,756	63
Blood glucose, mmol/l	7.91 (2.47)	30,415	54
eGFR, ml/min per 1.73 m^2^	88.39 (24.65)	30,916	54
Systolic BP, mmHg	131.67 (18.33)	31,933	57
Diastolic BP, mmHg	78.67 (11.3)	31,933	57
BMI, kg/m^2^	30.71 (8.11)	33,411	61
Total cholesterol, mmol/l	4.51 (1.29)	24,900	44
LDL-cholesterol, mmol/l	2.49 (1.13)	24,900	44
HDL-cholesterol, mmol/l	1.18 (0.38)	24,900	44
Triglycerides, mmol/l	1.6 (1.09)	24,900	44
Total insulin dose, U/day	31.93 (36.45)	31,206	55
Insulin origin			
Human	2048 (7.15)	28,650	62
Analogue	25,321 (88.38)	28,650	62
Human analogue	421 (1.47)	28,650	62
Insulin regimen			
Premix	3858 (13.72)	28,114	61
Basal bolus	8331 (29.63)	28,114	61
Basal	15,925 (56.64)	28,114	61
Concomitant disease at baseline			
CVD	21,784 (69.59)	31,302	59
Retinopathy	4378 (14.72)	29,745	56
Neuropathy	5326 (18.51)	28,770	53
Nephropathy	2597 (9.29)	27,942	49
Concomitant medication			
Psychoactive drug	4396 (13.63)	32,252	61
Glucose-lowering drug	24,226 (78.24)	30,965	58
Anti-epileptic drug	999 (3.16)	31,664	59
Anti-inflammatory drug	7529 (23.35)	32,241	60
Antithyroid drug	84 (0.33)	25,535	43
Antihypertensive drug	21,101 (65.43)	32,252	61
Antibiotic	220 (0.8)	27,408	51
Sex hormone	640 (2.01)	31,880	58

Median and IQR are reported for continuous variables; IQR is given as the distance between the 25th and 75th percentile; frequency and percentage are reported for categorical variables

ESM Tables 3–8 show the covariates considered for inclusion in multivariate prediction models, including those specifically included for each hypoglycaemia classification.

### Hypoglycaemia event rates

Rates of hypoglycaemia varied between trials, as shown for each hypoglycaemia level in ESM Figs 1, 2. For example, for type 1 diabetes, of trials where a level 3 hypoglycaemia event was recorded, the median hypoglycaemia event rate was 20.5 events/100 participants per year and the highest rate was 409 events/100 participants per year. For type 2 diabetes, the median level 3 hypoglycaemia event rate was 4.8 events/100 participants per year and the highest rate was 850.7 events/100 participants per year (individuals from this trial did not contribute to further analyses for level 3 outcomes; the next highest rate was 186.7 events/100 participants per year). Due to this high variability between trials all analyses were adjusted by trial.

### Minimally adjusted associations with hypoglycaemia

For type 1 diabetes, the number of people with available data ranged between 7613 and 12,247. Power was greatest to detect associations with total events overall since the numbers of level 2 and level 3 hypoglycaemia events were much lower than for level 1. As shown in Table 3, the following were associated with greater frequency of hypoglycaemia overall (level 1 or worse) and with at least a consistent direction of effect for the more severe levels; female sex; longer diabetes duration; lower HbA_1c_; greater self-monitored glucose and glucose variability; higher score for hypoglycaemia across the 6 week baseline period; using human rather than analogue only insulin; lower BMI; lower diastolic BP; lower total cholesterol; lower triglycerides; and use of anti-inflammatory and psychoactive drugs. Black or African American ethnicity was associated with fewer hypoglycaemia events than White ethnicity. Those with more complications had fewer hypoglycaemic events, though for level 3 the data were too sparse and the directions inconsistent with that for events overall. A lower insulin dose at the end of the baseline period was associated with more subsequent hypoglycaemia events in this minimally adjusted analysis.

**Table 3:** Type 1 diabetes minimally adjusted associations of baseline covariates with hypoglycaemia events across the trial duration

Covariate	Level 1 or worse hypoglycaemia RR (95% CI)	Level 2 or worse hypoglycaemia RR (95% CI)	Level 3 hypoglycaemia RR (95% CI)
Age, years	1.032 (1.000, 1.064)	0.974 (0.940, 1.009)	0.834 (0.736, 0.945)*
Female sex	Reference	Reference	Reference
Male sex	0.814 (0.779, 0.850)*	0.811 (0.772, 0.852)*	0.735 (0.616, 0.878)*
Diabetes duration, years	1.129 (1.100, 1.159)*	1.215 (1.179, 1.251)*	1.644 (1.487, 1.818)*
Ethnicity			
Black or African American	0.683 (0.576, 0.809)*	0.723 (0.596, 0.878)*	0.941 (0.498, 1.780)
Other	1.035 (0.940, 1.139)	1.029 (0.923, 1.147)	1.426 (0.984, 2.068)
White	Reference	Reference	Reference
HbA_1c_, mmol/mol	0.865 (0.846, 0.885)*	0.877 (0.855, 0.900)*	0.935 (0.855, 1.023)
HbA_1c_, %	0.865 (0.846, 0.885)*	0.877 (0.855, 0.900)*	0.935 (0.855, 1.023)
Blood glucose, mmol/l^a^	1.018 (1.006, 1.031)*	1.040 (1.026, 1.055)*	1.092 (1.040, 1.146)*
Blood glucose variability^a^	1.049 (1.047, 1.051)*	1.060 (1.058, 1.063)*	1.054 (1.044, 1.064)*
Log_*e*_(total insulin dose), U/day	0.768 (0.730, 0.807)*	0.808 (0.763, 0.856)*	1.129 (0.919, 1.385)
Insulin origin			
Analogue	Reference	Reference	Reference
Human	1.267 (0.988, 1.626)	1.184 (0.896, 1.563)	^b^
Human analogue	1.247 (1.055, 1.474)*	1.165 (0.967, 1.403)	1.420 (0.826, 2.442)
Previous hypoglycaemia score	1.123 (1.117, 1.128)*	1.163 (1.157, 1.169)*	1.161 (1.138, 1.185)*
Log_*e*_(eGFR), ml/min per 1.73 m^2^	1.046 (0.901, 1.202)	1.028 (0.877, 1.206)	1.017 (0.610, 1.696)
Systolic BP, mmHg	0.994 (0.992, 0.996)*	0.995 (0.993, 0.997)*	1.004 (0.997, 1.011)
Diastolic BP, mmHg	0.989 (0.987, 0.992)*	0.990 (0.987, 0.993)*	0.990 (0.980, 1.001)
BMI, kg/m^2^	0.966 (0.961, 0.972)*	0.965 (0.958, 0.971)*	0.986 (0.964, 1.009)
HDL-cholesterol, mmol/l	1.001 (1.000, 1.002)	1.001 (1.000, 1.002)	1.003 (1.000, 1.006)
LDL-cholesterol, mmol/l	1.000 (0.999, 1.000)	1.000 (0.999, 1.000)	0.998 (0.996, 1.001)
Total cholesterol, mmol/l	0.924 (0.900, 0.948)*	0.933 (0.905, 0.962)*	0.944 (0.841, 1.059)
Triglycerides, mmol/l	0.758 (0.732, 0.785)*	0.730 (0.699, 0.762)*	0.795 (0.666, 0.950)*
Concomitant disease at baseline			
CVD	0.807 (0.763, 0.853)*	0.807 (0.758, 0.860)*	1.113 (0.898, 1.381)
Retinopathy	0.893 (0.833, 0.957)*	0.847 (0.783, 0.917)*	0.916 (0.703, 1.194)
Neuropathy	0.863 (0.799, 0.932)*	0.886 (0.812, 0.967)*	1.176 (0.887, 1.560)
Nephropathy	0.808 (0.722, 0.905)*	0.792 (0.697, 0.902)*	1.018 (0.662, 1.566)
Concomitant medication^c^			
Anti-epileptic drug	0.950 (0.811, 1.112)	1.073 (0.897, 1.283)	1.857 (1.099, 3.138)*
Antihypertensive drug	0.813 (0.768, 0.861)*	0.807 (0.757, 0.861)*	1.082 (0.871, 1.344)
Anti-inflammatory drug	1.287 (1.221, 1.357)*	1.292 (1.217, 1.371)*	1.315 (1.074, 1.609)*
Psychoactive drug	1.038 (0.964, 1.118)	1.115 (1.026, 1.212)*	1.511 (1.168, 1.955)*
Sex hormone	1.104 (0.995, 1.224)	1.054 (0.937, 1.184)	0.775 (0.517, 1.161)
Steroid cessation	0.991 (0.366, 2.682)	0.945 (0.304, 2.936)	3.117 (0.222, 43.752)

For continuous covariates, data represent the increase in hypoglycaemia rate for every SD change in covariate for the first 6 weeks of the study; for categorical covariates, data represent the increase in hypoglycaemia rate with respect to the reference category. In both cases, adjusted for age, sex, diabetes duration and study identifier as fixed effects, and individual identifier as random effect

^a^Blood glucose was determined by self-monitoring

^b^Only a single type 1 diabetes study contained human only insulin, and this study recorded no severe hypoglycaemia events and so no association was estimated in this case

^c^Association with antibiotics, antithyroid and glucose-lowering medications were excluded from this analysis as numbers of observations were low

^*^p<0.05 (associations where the CI does not cross 1)

For type 2 diabetes, the number of people with available covariate data ranged between 24,900 and 34,007. As shown in Table 4, the following covariates were associated with more hypoglycaemia events overall (level 1 or worse) and with at least a consistent direction of effect for more severe levels: older age; female sex; longer diabetes duration; more glucose variability; higher score for hypoglycaemia across the 6 week baseline period; use of human rather than analogue only insulin; use of premix or basal bolus rather than basal insulin; lower eGFR; lower systolic and diastolic BP; lower total cholesterol; lower triglycerides; using concomitant oral glucose-lowering drugs; and exposure to other drug classes including psychoactive drugs. Black ethnicity, African American ethnicity and ethnicity other than White was associated with fewer hypoglycaemia events than White ethnicity.

**Table 4:** Type 2 diabetes minimally adjusted associations of baseline covariates with hypoglycaemia events across the trial duration

Covariate	Level 1 or worse hypoglycaemia RR (95% CI)	Level 2 or worse hypoglycaemia RR (95% CI)	Level 3 hypoglycaemia RR (95% CI)
Age, years	1.135 (1.111, 1.159)*	1.049 (1.018, 1.080)*	1.092 (0.967, 1.234)
Female sex	Reference	Reference	Reference
Male sex	0.899 (0.864, 0.934)*	0.881 (0.835, 0.929)*	0.742 (0.591, 0.933)*
Diabetes duration, years	1.258 (1.231, 1.285)*	1.257 (1.222, 1.294)*	1.21 (1.077, 1.359)*
Ethnicity			
Black or African American	1.037 (0.950, 1.132)	1.037 (0.920, 1.170)	0.840 (0.515, 1.369)
Other	0.830 (0.771, 0.894)*	0.726 (0.652, 0.807)*	0.445 (0.267, 0.744)*
White	Reference	Reference	Reference
HbA_1c_, mmol/mol	0.940 (0.921, 0.961)*	0.971 (0.943, 1.000)	1.026 (0.911, 1.156)
HbA_1c_, %	0.940 (0.921, 0.961)*	0.971 (0.943, 1.000)	1.026 (0.911, 1.156)
Blood glucose, mmol/l	0.944 (0.934, 0.954)*	0.991 (0.977, 1.006)	1.118 (1.058, 1.182)*
Blood glucose variability	1.057 (1.055, 1.059)*	1.065 (1.062, 1.068)*	1.053 (1.040, 1.067)*
Log_*e*_(total insulin dose), U/day	0.985 (0.947, 1.025)	1.003 (0.951, 1.059)	1.185 (0.945, 1.485)
Insulin origin^a^			
Analogue	Reference	Reference	Reference
Human	1.641 (1.352, 1.991)*	1.865 (1.461, 2.381)*	5.486 (0.423, 71.125)
Human analogue	1.171 (0.862, 1.590)	1.203 (0.839, 1.725)	1.737 (0.110, 27.549)
Insulin regimen			
Basal bolus	1.595 (1.283, 1.984)*	2.315 (1.698, 3.157)*	0.702 (0.140, 3.532)
Premix	1.190 (1.022, 1.386)*	1.814 (1.463, 2.249)*	0.657 (0.140, 3.075)
Basal	Reference	Reference	Reference
Previous hypoglycaemia score	1.406 (1.383, 1.429)*	1.553 (1.525, 1.583)*	1.366 (1.283, 1.455)*
Log_*e*_(eGFR), ml/min per 1.73 m^2^	0.800 (0.723, 0.886)*	0.776 (0.675, 0.893)*	0.795 (0.459, 1.378)
Systolic BP, mmHg	0.997 (0.996, 0.998)*	0.997 (0.995, 0.999)*	0.988 (0.979, 0.996)*
Diastolic BP, mmHg	0.987 (0.985, 0.990)*	0.987 (0.984, 0.990)*	0.980 (0.966, 0.993)*
BMI, kg/m^2^	0.962 (0.959, 0.966)*	0.961 (0.956, 0.966)*	1.008 (0.986, 1.029)
HDL-cholesterol, mmol/l	1.003 (1.002, 1.004)*	1.003 (1.002, 1.004)*	1.004 (0.995, 1.012)
LDL-cholesterol, mmol/l	1.000 (0.999, 1.000)	1.000 (0.999, 1.000)	0.993 (0.987, 0.999)*
Total cholesterol, mmol/l	0.923 (0.904, 0.942)*	0.914 (0.888, 0.941)*	0.810 (0.700, 0.937)*
Triglycerides, mmol/l	0.895 (0.880, 0.910)*	0.858 (0.836, 0.881)*	0.904 (0.788, 1.037)
Concomitant disease at baseline			
CVD	0.816 (0.776, 0.859)*	0.836 (0.779, 0.898)*	2.223 (1.492, 3.314)*
Retinopathy	0.973 (0.910, 1.040)	0.903 (0.823, 0.991)*	1.097 (0.702, 1.716)
Neuropathy	0.917 (0.864, 0.972)*	0.930 (0.857, 1.009)	1.415 (0.996, 2.011)
Nephropathy	0.908 (0.825, 0.999)*	0.894 (0.780, 1.025)	1.293 (0.652, 2.562)
Concomitant medication^b^			
Anti-epileptic drug	1.207 (1.076, 1.353)*	1.309 (1.121, 1.529)*	2.918 (1.755, 4.850)*
Antihypertensive drug	0.910 (0.869, 0.953)*	0.926 (0.868, 0.987)*	1.680 (1.215, 2.324)*
Anti-inflammatory drug	1.294 (1.233, 1.357)*	1.302 (1.219, 1.391)*	2.102 (1.613, 2.738)*
Glucose-lowering drug	1.029 (0.955, 1.109)	0.964 (0.874, 1.063)	0.810 (0.564, 1.163)
Psychoactive drug	1.252 (1.183, 1.324)*	1.352 (1.252, 1.459)*	3.041 (2.293, 4.034)*
Sex hormone	1.365 (1.193, 1.561)*	1.132 (0.944, 1.357)	1.808 (0.908, 3.603)
Steroid cessation	0.587 (0.252, 1.367)	0.463 (0.125, 1.723)	2.832 (0.122, 65.959)

For continuous covariates data represent the increase in hypoglycaemia rate for every SD change in covariate for the first 6 weeks of the study; for categorical covariates data represent the increase in hypoglycaemia rate with respect to the reference category. In both cases adjusted for age, sex, diabetes duration and study identifier as fixed effects, and individual identifier as random effect

^a^Level 3 events were low in human+analogue insulin leading to wide CIs when estimating associations with insulin origin

^b^Association with antibiotics and antithyroid medications were excluded from this analysis as number of observations were low. Blood glucose is self-monitored

^*^p<0.05 (associations where the CI does not cross 1)

Thus the main difference in direction in these minimally adjusted associations when comparing type 1 diabetes with type 2 diabetes were associations with more of the examined drug classes in type 2 diabetes where such concomitant use was more frequent. Since these associations were minimally adjusted, they were descriptive rather than necessarily indicative of causality.

See ESM Tables 9 and 10 for associations between risk factors and hypoglycaemia where missing data was not imputed.

### Prediction modelling

Here the goal was to evaluate how risk factor status at baseline predicted the hypoglycaemia risk across the duration of the trial. The final numbers of individuals in training and testing after exclusion for missingness are shown in Table 5.

**Table 5:** Number of individuals in each data partition for multivariate analysis after all data exclusion for multivariate analysis

Data partition	Type 1 diabetes			Type 2 diabetes		
	Level 1 or worse hypoglycaemia	Level 2 or worse hypoglycaemia	Level 3 hypoglycaemia	Level 1 or worse hypoglycaemia	Level 2 or worse hypoglycaemia	Level 3 hypoglycaemia
Train	5911	5911	4927	13,645	13,645	5846
Test	2523	2523	2102	5827	5827	2500
Total	8434	8434	7029	19,472	19,472	8346

Prediction of any hypoglycaemia event of any severity was greater than for more sparse events requiring assistance (level 3 hypoglycaemia) in both types of diabetes (Tables 6, 7). For prediction of level 1 or worse hypoglycaemia during the whole follow-up period, the AUC with the full model was 0.835 (0.826, 0.844) in type 1 diabetes and was 0.840 (0.831, 0.848) in type 2 diabetes. For level 3 hypoglycaemia the AUC was lower, at 0.689 (0.667, 0.712) for type 1 diabetes and 0.705 (0.662, 0.748) for type 2 diabetes. As shown in Tables 6 and 7, the increment in AUC compared with the baseline model was substantial when the score for hypoglycaemia in the 6 week baseline phase was included with the other covariates in the full model, contributing a little more. This was confirmed by the XGBoost importance procedure on the full models shown in Tables 8 and 9. The calculated ‘gain’ measure from this procedure captured the relative importance of inclusion of a given covariate to improving model fit.

**Table 6:** Type 1 diabetes test performance of hypoglycaemia event XGBoost prediction models using covariates evaluated over a 6 week baseline and hypoglycaemia events thereafter

Model	Level 1 or worse hypoglycaemia (test: 2523 people)		Level 2 or worse hypoglycaemia (test: 2523 people)		Level 3 hypoglycaemia (test: 2102 people)	
	AUC	LL	AUC	LL	AUC	LL
Baseline	0.674 (0.661, 0.687)	0.0	0.612 (0.597, 0.626)	0.0	0.625 (0.601, 0.648)	0.0
Baseline with previous hypoglycaemia	0.827 (0.818, 0.836)	56,025.2	0.825 (0.815, 0.835)	32,593.6	0.701 (0.68, 0.723)	1198.4
Full	0.835 (0.826, 0.844)	60,554.0	0.812 (0.801, 0.822)	31,250.4	0.689 (0.667, 0.712)	892.7

AUC refers to the AUC for the receiver operating characteristic curve on the test dataset with 95% CIs in parentheses

LL, change in test log_*e*_(likelihood) from baseline model

**Table 7:** Type 2 diabetes test performance of hypoglycaemia event XGBoost prediction models using covariates evaluated over a 6 week baseline and hypoglycaemia events thereafter

Model	Level 1 or worse hypoglycaemia (test: 2523 people)		Level 2 or worse hypoglycaemia (test 2523 people)		Level 3 hypoglycaemia (test: 2102 people)	
	AUC	LL	AUC	LL	AUC	LL
Baseline	0.671 (0.659, 0.682)	0.0	0.774 (0.764, 0.784)	0.0	0.570 (0.525, 0.616)	0.0
Baseline with previous hypoglycaemia	0.810 (0.801, 0.819)	27,015.0	0.842 (0.833, 0.851)	4124.1	0.690 (0.649, 0.731)	130.2
Full	0.840 (0.831, 0.848)	30,793.6	0.866 (0.858, 0.874)	4936.3	0.705 (0.662, 0.748)	166.2

AUC refers to the AUC for the receiver operating characteristic curve on the test dataset with 95% CIs in parentheses

LL, change in test log_*e*_(likelihood) from baseline model

**Table 8:** Type 1 diabetes covariate importance for hypoglycaemia event prediction over follow-up when covariates are evaluated with a 6 week interval following randomisation as determined by XGBoost importance gain measure

Level 1 or worse hypoglycaemia		Level 2 or worse hypoglycaemia		Level 3 hypoglycaemia	
Feature	Gain	Feature	Gain	Feature	Gain
Previous hypoglycaemia	0.51	Previous hypoglycaemia	0.42	Age	0.28
Self-monitored blood glucose	0.06	Self-monitored blood glucose	0.08	Previous hypoglycaemia	0.19
HbA_1c_	0.04	HbA_1c_	0.06	eGFR	0.11
Total daily insulin dose	0.04	Self-monitored blood glucose variability	0.06	Self-monitored blood glucose	0.08
Self-monitored blood glucose variability	0.04	Diabetes duration	0.06	BMI	0.07
Diabetes duration	0.04	Age	0.05	Self-monitored blood glucose variability	0.07
eGFR	0.04	Total daily insulin dose	0.05	Diabetes duration	0.05
Age	0.04	eGFR	0.05	Total daily insulin dose	0.03
BMI	0.03	BMI	0.05	Systolic BP	0.03
Systolic BP	0.03	Systolic BP	0.03	HbA_1c_	0.03

**Table 9:** Type 2 diabetes covariate importance for hypoglycaemic event prediction over follow-up when covariates are evaluated with a 6 week interval following randomisation as determined by XGBoost importance gain measure

Level 1 or worse hypoglycaemia		Level 2 or worse hypoglycaemia		Level 3 hypoglycaemia	
Feature	Gain	Feature	Gain	Feature	Gain
Previous hypoglycaemia	0.27	Previous hypoglycaemia	0.4	Previous hypoglycaemia	0.15
Self-monitored blood glucose variability	0.1	Self-monitored blood glucose variability	0.1	Self-monitored blood glucose variability	0.12
Self-monitored blood glucose	0.07	Total daily insulin dose	0.07	Self-monitored blood glucose	0.12
Diabetes duration	0.06	Self-monitored blood glucose	0.07	Age	0.08
Total daily insulin dose	0.06	Diabetes Duration	0.04	eGFR	0.08
BMI	0.05	BMI	0.04	Diabetes duration	0.07
Age	0.05	SBP	0.04	HbA_1c_	0.06
SBP	0.05	Age	0.04	Total daily insulin dose	0.06
eGFR	0.05	eGFR	0.04	Systolic BP	0.06
HbA_1c_	0.05	HbA_1c_	0.04	BMI	0.04
Insulin Regimen: Basal bolus	0.03	Insulin regimen: basal bolus Insulin regimen: premix	0.02 0.02	Retinopathy at baseline Insulin origin: human Sex	0.02 0.02 0.01

## Discussion

The Hypo-RESOLVE database brought together a large volume of prospective data on hypoglycaemia in type 1 and type 2 diabetes from clinical trials of glucose-lowering agents. The dataset is one of the largest reported to date for examining risk factors and prediction of hypoglycaemia. We show that many factors associated with having greater frequency of hypoglycaemia are common to type 1 and type 2 diabetes. Of particular note is a consistent finding that there is a higher rate of hypoglycaemia in the female sex than in the male sex for both type of diabetes. Ethnic differences were also observed that deserve further exploration and confirmation in other datasets. In both type 1 and type 2 diabetes a higher rate of hypoglycaemia was observed in individuals using human insulin, either alone or combined, than in those using analogue only insulin.

More drug categories were associated with more hypoglycaemia in type 2 diabetes than in type 1 diabetes. Furthermore, lower eGFR was associated with greater frequency of hypoglycaemia in type 2 diabetes. In both types of diabetes use of psychotropic drugs was associated with more hypoglycaemia events suggesting that careful consideration of the need for such drugs is warranted in those experiencing frequent or severe hypoglycaemia. Greater self-monitored glucose variability and more recent hypoglycaemia were also associated with the prospective risk of hypoglycaemia in both type 1 and type 2 diabetes. An association between lower insulin dose at the end of the baseline data collection period of weeks 1 to 6 and subsequent hypoglycaemia may be expected due to dose adjustments in response to a higher incidence of hypoglycaemia events during this period.

The multivariate analyses showed that future hypoglycaemia risk was highly predictable and that almost all of this prediction is obtainable from an individual’s recent hypoglycaemia history rather than these other associated characteristics. These data suggest that a formal prediction model would be unnecessary since recent history of hypoglycaemia is usually available from patients and their healthcare professionals. However, our data provide validation of the methodology and highlight potentially modifiable risk factors, including choice of insulin and concurrent medications, that should be considered in attempts to ameliorate hypoglycaemia risk.

### Comparison with existing literature

Our study differs from many in being larger, involving a population drawn from clinical trials and, importantly, using a consistent set of definitions of hypoglycaemia. Our data replicate some well-recognised associations for hypoglycaemia, validating our methodology, such as worse hypoglycaemia risk with longer diabetes duration [15] and less frequent hypoglycaemia with insulin analogue [16–20]. Associations with less frequent hypoglycaemia with insulin in our study is not explained by differences in diabetes duration since within trial duration was similar, reflecting the randomisation, and since our method of analysis is akin to summarising the within-trial associations across all trials. The link between impaired renal function in type 2 diabetes is also widely reported [21, 22]. Links between polypharmacy and increased hypoglycaemia risk in people with type 2 diabetes are well described and may be driven both by increasing frailty and interactions between therapeutic agents themselves [23]. The link with non-steroidal anti-inflammatory drug use is interesting. Some drugs of this class are recognised to cause hypoglycaemia through a mechanism that may involve modulation of ion channel activities, either in the pancreatic beta cell or central nervous system glucose-sensing neurons [24]. Our data are consistent with reports of increased hypoglycaemia risk for the female sex in cohorts including both diabetes types [25, 26] and in type 2 diabetes cohort s [27–30], although, conversely, a small number of studies of cohorts including both diabetes types report the male sex to be at increased risk of hypoglycaemia [31–33]. A possible explanation for increased hypoglycaemia risk for the female sex is that they have less pronounced counterregulatory response during experimental hypoglycaemia than the male sex [34]. There could also be a differential distribution between sexes in other unmeasured confounders, such as comorbidities or concurrent medications, that could affect hypoglycaemia risk. Other factors determining trial participation (e.g. socioeconomic status) may confound the ethnicity association we found and thus this warrants further investigation.

It is worth noting that in both types of diabetes greater glucose variability was clearly a risk factor for more frequent hypoglycaemia. The existing literature supports an association between increased glucose variability and increased hypoglycaemia risk [35, 36]. However, the literature for HbA_1c_ and hypoglycaemia risk is controversial as multiple studies report an association between low HbA_1c_ and increased risk [27, 37–39], and multiple studies report no association [40–44]. A 6 week history of increased hypoglycaemia frequency was the most important predictive factor, as expected, and is consistent with cohorts including both types of diabetes [45–48] and type 2 diabetes cohort s [40, 48–50].

We found rates of level 3 hypoglycaemia that were lower than US-based estimates from ambidirectional panel survey data [51] but higher than estimates from Scotland for rates of hypoglycaemia requiring emergency medical treatment [52]

### Limitations of this analysis

The use of trial data allowed us to bring together a large dataset wherein hypoglycaemia of differing severity level was well characterised and captured, unlike in many population studies. However, it also presented several challenges. A substantial challenge was that the trials represented very different subpopulations, with prior severe hypoglycaemia within 12 months being an exclusion criteria in many trials. Widely varying incidence rates for all levels of hypoglycaemia necessitated the study number being required as a covariate in the analysis to avoid confounding by study entry criteria. Absolute rates of hypoglycaemia within these trials cannot be generalised, although useful information on predictors of hypoglycaemia remain despite the intervention setting of these trials. Furthermore while we had good capture of many important variables, several variables that would be of interest to examine in relation to hypoglycaemia, such as C-peptide levels in type 1 diabetes [42], were not available in these trials. There was a high level of missingness for some variables requiring imputation. Other variables, such as late diabetic complication status, were only crudely assessed as present/absent and not all trials routinely captured all concurrent medications.

Finally, trial entrants were not necessarily representative of the general population living with diabetes and, while this limits the generalisability of the observed incidence rates of hypoglycaemia and sociodemographic features that might be confounded by association with other determinants of hypoglycaemia, it is likely that among trial participants the relationship between most clinical characteristics and hypoglycaemia is preserved.

### Conclusions

The key findings from this large study are generalisable: a small number of variables along with recent hypoglycaemia rate over a 6 week period provide the level of prediction of future hypoglycaemia at varying levels of severity that would be useful in several settings; and other associated clinical characteristics add little to prediction over the time horizon we studied. Such settings might include selection of people with diabetes into clinical trials, raising alerts as to high risks that require immediate mitigation. or prioritising those to be given scarce resources such as CGM or semi-closed/closed loop systems. Finally, the data reinforce our view that careful capture and consideration of recent hypoglycaemia history and review of glucose monitoring data and modifiable risk factors are fundamental to providing good clinical care in diabetes.

### Supplementary Information

Below is the link to the electronic supplementary material.

ESM 1 (PDF 259 KB)

## Data Availability

The data underlying the results presented in the study come from the Hypo-RESOLVE data repository, which will be maintained for a 2 year period following the end of the Hypo-RESOLVE project. Enquiries about third-party researcher data access and associated access criteria should be sent to the Hypo-RESOLVE data access committee (Jakob Haardt J.Haardt@eurice.eu / Mark Ibberson Mark.Ibberson@sib.swiss; or via https://hypo-resolve.eu/contact).

## Abbreviations

CGM: Continuous glucose monitoring
GLMM: Generalised linear mixed model
Hypo-RESOLVE: Hypoglycaemia REdefining SOLutions for better liVEs

## Authors’ relationships and activities

HMC has research grants from JDRF and Diabetes UK. She has participated in a speakers bureau, on an advisory board for Novo Nordisk, on an advisory panel for Bayer AG, and is a shareholder of Roche Pharmaceuticals and Bayer AG. SH has participated on advisory boards for Eli Lilly and Novo Nordisk and on a speakers bureau for Novo Nordisk and has received honorarium via his institution from Medtronic. He has consulted for Vertex Pharma, Zucara Pharmaceuticals and Zealand Pharma. AS is an employee of, and holds shares in, Novo Nordisk. UP-B received personal honorarium for lectures, presentations and expert testimony from Sanofi and Novo Nordisk and is a member of the editorial board of *Diabetologia*. BEdG is a member of the editorial board of *Diabetologia*. RJM received personal honorarium for lectures, and presentations from Sanofi and Novo Nordisk, and support for travel to lecture at an educational event from Sanofi. He is a non-executive member of NHS Tayside Health Board. MR is an employee of, and holds shares in, Eli Lilly and Company. SAA has served on Advisory Boards for Vertex Pharmaceuticals and spoken at educational events sponsored by Sanofi and Novo Nordisk. M-AG is an employee of, and holds shares in, Novo Nordisk. All other authors declare that there are no relationships or activities that might bias, or be perceived to bias, their work.
