# Supplementary material for: Risk factors and prediction of hypoglycaemia using the Hypo-RESOLVE cohort: a secondary analysis of pooled data from insulin clinical trials
Source: Diabetologia. 2024 May 25;67(8):1588–601. doi: 10.1007/s00125-024-06177-6 (PMC11343909; doi:10.1007/s00125-024-06177-6)
Supplement: Supplementary file 1 — ESM 1 (PDF 259 KB) [file 125_2024_6177_MOESM1_ESM.pdf]

Supplementary material for Risk factors and prediction of hypoglycaemia using the Hypo-RESOLVE cohort: a secondary analysis of pooled data from insulin clinical trials

Joseph Mellor[1] Dmitry Kuznetsov[2] Simon Heller[12] Mari-Anne Gall[9]
Myriam Rosilio[10] Stephanie A. Amiel[11] Mark Ibberson[2]
Stuart McGurnaghan[3] Luke Blackbourn[3] William Berthon[1] Adel Salem[4]
Yongming Qu[5] Rory J. McCrimmon[6] Bastiaan E. de Galan[7]
Ulrik Pedersen-Bjergaard[8] Joanna Leaviss[13] Paul M. McKeigue[1]
Helen M. Colhoun[3]

Contents

1 Methods **2**
1.1 CDISC domains and tables 2
1.2 Medical history definitions 2
1.3 Concomitant Medications definitions 2
1.4 XGBoost 2

1. Usher Institute, College of Medicine and Veterinary Medicine, University of Edinburgh, Teviot Place, Edinburgh, EH8 9AG, UK
2. Swiss Institute of Bioinformatics, Lausanne, Switzerland
3. Institute of Genetics and Cancer, College of Medicine and Veterinary Medicine, University of Edinburgh, Western General Hospital Campus, Crewe Road, Edinburgh EH4 2XUC, UK
4. RW Data Assets, AI & Analytics (AIA), Novo Nordisk A/S, Soeberg, Denmark
5. Eli Lilly and Company, Indianapolis, United States
6. Systems Medicine, School of Medicine, University of Dundee, Dundee, UK
7. Department of Internal Medicine, Division of Endocrinology and Metabolic Disease, Maastricht University Medical Center, Maastricht, the Netherlands
8. Institute of Clinical Medicine, University of Copenhagen, Copenhagen, Denmark
9. Medical & Science, Insulin, Clinical Drug Development, Novo Nordisk A/S, Soeberg, Denmark
10. Eli Lilly and Company, Diabetes Medical Unit, Neuilly sur seine, France
11. Department of Diabetes, School of Cardiovascular and Metabolic Medicine and Sciences, Faculty of Life Sciences and Medicine, King's College London, London, UK
12. Division of Clinical Medicine, University of Sheffield, Sheffield, UK
13. School of Health and Related Research (ScHARR), University of Sheffield, Sheffield, UK

1 Methods

1.1 CDISC domains and tables

The data were organised in tables/domains including AE (adverse events), DM (demographics), EX (treatment exposure), VS (vital signs), SV (subject visits), MH (medical history), CM (concomitant medications), LB (laboratory measurements), TA (trial arms), SE (subject elements).

1.2 Medical history definitions

Medical history events of interest were defined as shown in ESM Table 1.

1.3 Concomitant Medications definitions

Concomitant medications were defined as shown in ESM Table 2.

1.4 XGBoost

A XGBoost model forms its prediction as the sum of a number of predictive trees where each tree provides a given prediction via a series of sequential decision rules with each decision rule based on a single covariate. The result of a decision rule in a tree determines which decision rule is applied next to the data. The process continues until the last rule in the given sequence is reach and a predictive value conditioned on the decisions is given. When fitting a decision tree, a new decision rule is only added to the tree if it provides sufficient predictive gain. Given a covariate can appear in multiple decision rules within a single tree and can appear in multiple trees, the average predictive gain of all decision rules using the covariate is used as a measure of the importance of that covariate.
XGBoost easily fits non-linear effects of covariates and interaction effects between covariates without requiring explicit user-intervention in modelling. Due to differences in study populations it is reasonable to expect that effect sizes of common risk factors may be different within different trials for example. However, XGBoost models each 6 week time slice for an individual as independent.

Grid search was performed of the following parameters of XGBoost: eta (values: 1.0, 0.1, 0.01, and 0.001), gamma (values: 1.0, 0.1, and 0.001), and max_depth (values: 2, 4, 8, and 16).

ESM Table 1: Medical History definitions

Condition	Description
Retinopathy	'Diabetic Retinopathy' MEDDRA preferred term in the MH table
Neuropathy	'Diabetic Neuropathy' MEDDRA preferred term in the MH table
Nephropathy	'Diabetic Nephropathy' MEDDRA preferred term in the MH table
CVD	MEDDRA preferred terms in the MH table corresponding to the following standardised MEDDRA queries: Cardiac arrhythmias (SMQ); Cardiac failure (SMQ); Cardiomyopathy (SMQ); Central nervous system vascular disorders (SMQ); Embolic and thrombotic events (SMQ); Haemodynamic oedema, effusions and fluid overload (SMQ); Hypertension (SMQ); Ischaemic heart disease (SMQ); Pulmonary hypertension (SMQ); Shock (SMQ); Torsade de pointes/QT prolongation (SMQ)

ESM Table 2: Concomitant Medications definitions

Concomitant Medication	Description
Sex hormones drugs	ATC level 2 codes for Sex hormones and modulators of the genital system
Anti-epileptic drugs	ATC level 2 codes for anti-epileptics (N03)
Anti-thyroid drugs	ATC level 3 codes for antithyroid preparations (H03B)
Steroids	ATC level 2 codes for corticosteroids for systemic use (H02)
Psychoactive drugs	ATC level 3 codes for Antidepressants (N06A), and Opioids (N02A). ATC level 4 codes for Benzodiazepine derivatives (N05BA), and Antidepressants in combination with psycholeptics (N06CA). ATC level 5 codes for Cannabinoids (includes nabiximols) (N02BG10), Cocaine (N01BC01), and Haloperidol (N05AD01)
Glucose-lowering drugs	Blood-glucose lowering drugs excluding insulin ATC level 3 code A10B
Antihypertensive drugs	ATC level 2 codes for: Anti-hypertensives (C02), Diuretics (C03), Beta blocking agents (C07), Calcium channel blockers (C08), and agents acting on the renin-angiotensin system
Systemic Antibiotics	ATC level 3 codes for Quinolone antibacterials (JO1M) and ATC level 2 codes for Antiprotozoals (P01)
Systemic Oral Anti-inflammatory drugs	ATC level 2 codes for Anti-inflammatory and antirheumatic products (M01), ATC level 3 codes for Intestinal anti-inflammatory agents (A07E) and Other analgesics and antipyretics (N02B), and ATC level 4 codes for Anti-inflammatory products for vaginal administration (G02CC)

Note:
See https://www.who.int/tools/atc-ddd-toolkit/atc-classification
for Anatomical Therapeutic Chemical (ATC) classification

ESM Table 3: Candidate covariates for type 1 diabetes Level 1 or worse hypoglycaemic event analysis .

Step	Covariates
Full covariate set	Sex, Age, Diabetes duration, Study Identifier, Previous Hypos, HbA1c, Insulin regimen, Insulin origin, Insulin dose, eGFR, SBP, DBP, CVD at baseline, Retinopathy at baseline, Neuropathy at baseline, Nephropathy at baseline, Blood glucose, Blood glucose variability, Total Cholesterol, LDL Cholesterol, HDL Cholesterol, Triglycerides, BMI, Ethnicity, Antihypertensive drugs, Antibiotics, Anti-inflammatory drugs, Sex hormones, Psychoactive drugs, Anti-epileptic drugs, Anti-thyroid drugs, Steroid Cessation, Glucose-lowering drugs
After dropping covariates due to limited events or high inter-covariate colinearity	Sex, Age, Diabetes duration, Study Identifier, Previous Hypos, HbA1c, Insulin origin, Insulin dose, eGFR, SBP, Retinopathy at baseline, Blood glucose, Blood glucose variability, Total Cholesterol, LDL Cholesterol, HDL Cholesterol, Triglycerides, BMI, Ethnicity, Antihypertensive drugs, Anti-inflammatory drugs, Sex hormones, Psychoactive drugs
After removing covariates with a constant value or have more than 20% missingness	Sex, Age, Diabetes duration, Study Identifier, Previous Hypos, HbA1c, Insulin origin, Insulin dose, eGFR, SBP, Retinopathy at baseline, Blood glucose, Blood glucose variability, BMI, Ethnicity, Antihypertensive drugs, Anti-inflammatory drugs, Sex hormones, Psychoactive drugs

ESM Table 4: Candidate covariates for type 1 diabetes Level 2 or worse hypoglycaemic event analysis .

Step	Covariates
Full covariate set	Sex, Age, Diabetes duration, Study Identifier, Previous Hypos, HbA1c, Insulin regimen, Insulin origin, Insulin dose, eGFR, SBP, DBP, CVD at baseline, Retinopathy at baseline, Neuropathy at baseline, Nephropathy at baseline, Blood glucose, Blood glucose variability, Total Cholesterol, LDL Cholesterol, HDL Cholesterol, Triglycerides, BMI, Ethnicity, Antihypertensive drugs, Antibiotics, Anti-inflammatory drugs, Sex hormones, Psychoactive drugs, Anti-epileptic drugs, Anti-thyroid drugs, Steroid Cessation, Glucose-lowering drugs
After dropping covariates due to limited events or high inter-covariate colinearity	Sex, Age, Diabetes duration, Study Identifier, Previous Hypos, HbA1c, Insulin origin, Insulin dose, eGFR, SBP, Retinopathy at baseline, Blood glucose, Blood glucose variability, Total Cholesterol, LDL Cholesterol, HDL Cholesterol, Triglycerides, BMI, Ethnicity, Antihypertensive drugs, Anti-inflammatory drugs, Sex hormones, Psychoactive drugs
After removing covariates with a constant value or have more than 20% missingness	Sex, Age, Diabetes duration, Study Identifier, Previous Hypos, HbA1c, Insulin origin, Insulin dose, eGFR, SBP, Retinopathy at baseline, Blood glucose, Blood glucose variability, BMI, Ethnicity, Antihypertensive drugs, Anti-inflammatory drugs, Sex hormones, Psychoactive drugs

ESM Table 5: Candidate covariates for type 1 diabetes Level 3 hypoglycaemic event analysis .

Step	Covariates
Full covariate set	Sex, Age, Diabetes duration, Study Identifier, Previous Hypos, HbA1c, Insulin regimen, Insulin origin, Insulin dose, eGFR, SBP, DBP, CVD at baseline, Retinopathy at baseline, Neuropathy at baseline, Nephropathy at baseline, Blood glucose, Blood glucose variability, Total Cholesterol, LDL Cholesterol, HDL Cholesterol, Triglycerides, BMI, Ethnicity, Antihypertensive drugs, Antibiotics, Anti-inflammatory drugs, Sex hormones, Psychoactive drugs, Anti-epileptic drugs, Anti-thyroid drugs, Steroid Cessation, Glucose-lowering drugs
After dropping covariates due to limited events or high inter-covariate colinearity	Sex, Age, Diabetes duration, Study Identifier, Previous Hypos, HbA1c, Insulin origin, Insulin dose, eGFR, SBP, Retinopathy at baseline, Blood glucose, Blood glucose variability, Total Cholesterol, LDL Cholesterol, HDL Cholesterol, Triglycerides, BMI, Ethnicity, Antihypertensive drugs, Anti-inflammatory drugs, Sex hormones, Psychoactive drugs
After removing covariates with a constant value or have more than 20% missingness	Sex, Age, Diabetes duration, Study Identifier, Previous Hypos, HbA1c, Insulin origin, Insulin dose, eGFR, SBP, Retinopathy at baseline, Blood glucose, Blood glucose variability, BMI, Ethnicity, Antihypertensive drugs, Anti-inflammatory drugs, Sex hormones, Psychoactive drugs

ESM Table 6: Candidate covariates for type 2 diabetes Level 1 or worse hypoglycaemic event analysis .

Step	Covariates
Full covariate set	Sex, Age, Diabetes duration, Study Identifier, Previous Hypos, HbA1c, Insulin regimen, Insulin origin, Insulin dose, eGFR, SBP, DBP, CVD at baseline, Retinopathy at baseline, Neuropathy at baseline, Nephropathy at baseline, Blood glucose, Blood glucose variability, Total Cholesterol, LDL Cholesterol, HDL Cholesterol, Triglycerides, BMI, Ethnicity, Antihypertensive drugs, Antibiotics, Anti-inflammatory drugs, Psychoactive drugs, Anti-epileptic drugs, Anti-thyroid drugs, Sex hormones, Steroid Cessation, Glucose-lowering drugs
After dropping covariates due to limited events or high inter-covariate colinearity	Sex, Age, Diabetes duration, Study Identifier, Previous Hypos, HbA1c, Insulin regimen, Insulin origin, Insulin dose, eGFR, SBP, Retinopathy at baseline, Blood glucose, Blood glucose variability, Total Cholesterol, LDL Cholesterol, HDL Cholesterol, Triglycerides, BMI, Ethnicity, Antihypertensive drugs, Anti-inflammatory drugs, Psychoactive drugs, Glucose-lowering drugs
After removing covariates with a constant value or have more than 20% missingness	Sex, Age, Diabetes duration, Study Identifier, Previous Hypos, HbA1c, Insulin regimen, Insulin origin, Insulin dose, eGFR, SBP, Retinopathy at baseline, Blood glucose, Blood glucose variability, BMI, Ethnicity, Antihypertensive drugs, Anti-inflammatory drugs, Psychoactive drugs, Glucose-lowering drugs

ESM Table 7: Candidate covariates for type 2 diabetes Level 2 or worse hypoglycaemic event analysis .

Step	Covariates
Full covariate set	Sex, Age, Diabetes duration, Study Identifier, Previous Hypos, HbA1c, Insulin regimen, Insulin origin, Insulin dose, eGFR, SBP, DBP, CVD at baseline, Retinopathy at baseline, Neuropathy at baseline, Nephropathy at baseline, Blood glucose, Blood glucose variability, Total Cholesterol, LDL Cholesterol, HDL Cholesterol, Triglycerides, BMI, Ethnicity, Antihypertensive drugs, Antibiotics, Anti-inflammatory drugs, Psychoactive drugs, Anti-epileptic drugs, Anti-thyroid drugs, Sex hormones, Steroid Cessation, Glucose-lowering drugs
After dropping covariates due to limited events or high inter-covariate colinearity	Sex, Age, Diabetes duration, Study Identifier, Previous Hypos, HbA1c, Insulin regimen, Insulin origin, Insulin dose, eGFR, SBP, Retinopathy at baseline, Blood glucose, Blood glucose variability, Total Cholesterol, LDL Cholesterol, HDL Cholesterol, Triglycerides, BMI, Ethnicity, Antihypertensive drugs, Anti-inflammatory drugs, Psychoactive drugs, Glucose-lowering drugs
After removing covariates with a constant value or have more than 20% missingness	Sex, Age, Diabetes duration, Study Identifier, Previous Hypos, HbA1c, Insulin regimen, Insulin origin, Insulin dose, eGFR, SBP, Retinopathy at baseline, Blood glucose, Blood glucose variability, BMI, Ethnicity, Antihypertensive drugs, Anti-inflammatory drugs, Psychoactive drugs, Glucose-lowering drugs

ESM Table 8: Candidate covariates for type 2 diabetes Level 3 hypoglycaemic event analysis .

Step	Covariates
Full covariate set	Sex, Age, Diabetes duration, Study Identifier, Previous Hypos, HbA1c, Insulin regimen, Insulin origin, Insulin dose, eGFR, SBP, DBP, CVD at baseline, Retinopathy at baseline, Neuropathy at baseline, Nephropathy at baseline, Blood glucose, Blood glucose variability, Total Cholesterol, LDL Cholesterol, HDL Cholesterol, Triglycerides, BMI, Ethnicity, Antihypertensive drugs, Antibiotics, Anti-inflammatory drugs, Psychoactive drugs, Anti-epileptic drugs, Anti-thyroid drugs, Sex hormones, Steroid Cessation, Glucose-lowering drugs
After dropping covariates due to limited events or high inter-covariate colinearity	Sex, Age, Diabetes duration, Study Identifier, Previous Hypos, HbA1c, Insulin regimen, Insulin origin, Insulin dose, eGFR, SBP, Retinopathy at baseline, Blood glucose, Blood glucose variability, Total Cholesterol, LDL Cholesterol, HDL Cholesterol, Triglycerides, BMI, Ethnicity, Antihypertensive drugs, Anti-inflammatory drugs, Psychoactive drugs, Glucose-lowering drugs
After removing covariates with a constant value or have more than 20% missingness	Sex, Age, Diabetes duration, Study Identifier, Previous Hypos, HbA1c, Insulin regimen, Insulin origin, Insulin dose, eGFR, SBP, Retinopathy at baseline, Blood glucose, Blood glucose variability, BMI, Ethnicity, Antihypertensive drugs, Anti-inflammatory drugs, Psychoactive drugs, Glucose-lowering drugs

ESM Table 9: Type 1 diabetes minimally-adjusted associations of baseline covariates without imputation with hypoglycaemia events across the trial duration.

	Level 1 or worse	Level 2 or worse	Level 3
Covariate	RR (95% CI)	RR (95% CI)	RR (95% CI)
Age (years)	1.032 (1, 1.064)	0.974 (0.94, 1.009)	0.834 (0.736, 0.945)*
Sex: Female	Reference	Reference	Reference
Sex: Male	0.814 (0.779, 0.85)*	0.811 (0.772, 0.852)*	0.735 (0.616, 0.878)*
Diabetes duration (years)	1.129 (1.1, 1.159)*	1.215 (1.179, 1.251)*	1.644 (1.487, 1.818)*
Ethnicity: Black or African American	0.683 (0.576, 0.809)*	0.723 (0.596, 0.878)*	0.941 (0.498, 1.78)
Ethnicity: Other	1.035 (0.94, 1.139)	1.029 (0.923, 1.147)	1.426 (0.984, 2.068)
Ethnicity: White	Reference	Reference	Reference
HbA1c (%)	0.865 (0.846, 0.885)*	0.878 (0.855, 0.9)*	0.935 (0.855, 1.023)
Blood glucose (mmol/L)	1.018 (1.006, 1.031)*	1.04 (1.025, 1.054)*	1.093 (1.04, 1.147)*
Blood glucose variability	1.049 (1.047, 1.051)*	1.06 (1.058, 1.063)*	1.054 (1.044, 1.064)*
log Total Daily Insulin dose (U/day)	0.767 (0.729, 0.806)*	0.806 (0.761, 0.853)*	1.127 (0.918, 1.384)
Insulin origin: Analogue	Reference	Reference	Reference
Insulin origin: Human	1.267 (0.988, 1.626)	1.184 (0.896, 1.563)	
Insulin origin: Human+Analogue	1.247 (1.055, 1.474)*	1.165 (0.967, 1.403)	1.42 (0.826, 2.442)
log eGFR (mL/min/1.73m^2)	1.046 (0.91, 1.202)	1.034 (0.881, 1.212)	1.018 (0.61, 1.697)
Systolic BP (mmHg)	0.994 (0.992, 0.996)*	0.995 (0.993, 0.997)*	1.004 (0.997, 1.011)
Diastolic BP (mmHg)	0.989 (0.987, 0.992)*	0.99 (0.987, 0.993)*	0.99 (0.98, 1.001)
BMI (kg/m^2)	0.967 (0.961, 0.972)*	0.965 (0.958, 0.971)*	0.986 (0.964, 1.009)
HDL Cholesterol (mmol/L)	1.001 (1, 1.002)	1.001 (1, 1.002)	1.003 (1, 1.006)
LDL Cholesterol (mmol/L)	1 (0.999, 1)	1 (0.999, 1)	0.998 (0.996, 1.001)
Total Cholesterol (mmol/L)	0.924 (0.9, 0.948)*	0.933 (0.905, 0.962)*	0.944 (0.841, 1.059)
Triglycerides	0.757 (0.731, 0.784)*	0.729 (0.699, 0.762)*	0.79 (0.661, 0.945)*
CVD At Baseline	0.807 (0.763, 0.853)*	0.807 (0.758, 0.86)*	1.113 (0.898, 1.381)
Retinopathy At Baseline	0.893 (0.833, 0.957)*	0.847 (0.783, 0.917)*	0.916 (0.703, 1.194)
Neuropathy At Baseline	0.863 (0.799, 0.932)*	0.886 (0.812, 0.967)*	1.176 (0.887, 1.56)
Nephropathy At Baseline	0.808 (0.722, 0.905)*	0.792 (0.697, 0.902)*	1.018 (0.662, 1.566)
CM: Anti-epileptic	0.95 (0.811, 1.112)	1.073 (0.897, 1.283)	1.857 (1.099, 3.138)*
CM: Anti-hypertensives	0.813 (0.768, 0.861)*	0.807 (0.757, 0.861)*	1.082 (0.871, 1.344)
CM: Anti-inflammatories	1.287 (1.221, 1.357)*	1.292 (1.217, 1.371)*	1.315 (1.074, 1.609)*
CM: Psychoactives	1.038 (0.964, 1.118)	1.115 (1.026, 1.212)*	1.511 (1.168, 1.955)*
CM: Sex-hormones	1.104 (0.995, 1.224)	1.054 (0.937, 1.184)	0.775 (0.517, 1.161)
CM: Steroid cessation	0.991 (0.366, 2.682)	0.945 (0.304, 2.936)	3.117 (0.222, 43.752)

Note:

Association with Antibiotics, Anti-thyroid, and Blood glucose lowering medications were excluded from this analysis as numbers of observations were low.

Only a single type 1 diabetes study contained Human only insulin, and this study recorded no severe hypoglycaemia events and so no association was estimated in this case.

Blood glucose is self-monitoring blood glucose

For continuous covariates this is the increase in hypoglycaemia rate for every standard deviate change in covariate for the first 6 weeks of the study and for categorical covariates it is the increase in hypoglycaemia rate with respect to the reference category.

In both cases adjusted for age, sex, diabetes duration and study identifier as fixed effects, and individual identifier as random effect.

* Associations where the confidence interval does not cross 1, and are therefore significant ($P < 0.05$), are highlighted in bold.

ESM Table 10: Type 2 diabetes minimally-adjusted associations of baseline covariates without imputation with hypoglycaemia events across the trial duration.

	Level 1 or worse	Level 2 or worse	Level 3
Covariate	RR (95% CI)	RR (95% CI)	RR (95% CI)
Age (years)	1.135 (1.111, 1.159)*	1.049 (1.018, 1.08)*	1.092 (0.967, 1.234)
Sex: Female	Reference	Reference	Reference
Sex: Male	0.899 (0.864, 0.934)*	0.881 (0.835, 0.929)*	0.742 (0.591, 0.933)*
Diabetes duration (years)	1.258 (1.231, 1.285)*	1.257 (1.222, 1.294)*	1.21 (1.077, 1.359)*
Ethnicity: Black or African American	1.037 (0.95, 1.132)	1.037 (0.92, 1.17)	0.84 (0.515, 1.369)
Ethnicity: Other	0.83 (0.771, 0.894)*	0.726 (0.652, 0.807)*	0.445 (0.267, 0.744)*
Ethnicity: White	Reference	Reference	Reference
HbA1c (%)	0.941 (0.921, 0.961)*	0.971 (0.943, 1)	1.026 (0.91, 1.156)
Blood glucose (mmol/L)	0.945 (0.935, 0.955)*	0.99 (0.976, 1.005)	1.116 (1.056, 1.18)*
Blood glucose variability	1.057 (1.055, 1.059)*	1.065 (1.062, 1.068)*	1.052 (1.039, 1.065)*
log Total Daily Insulin dose (U/day)	0.978 (0.939, 1.019)	1.001 (0.946, 1.058)	1.204 (0.951, 1.523)
Insulin origin: Analogue	Reference	Reference	Reference
Insulin origin: Human	1.641 (1.352, 1.991)*	1.865 (1.461, 2.381)*	5.486 (0.423, 71.125)
Insulin origin: Human+Analogue	1.171 (0.862, 1.59)	1.203 (0.839, 1.725)	1.737 (0.11, 27.549)
Insulin Regimen: Basal-bolus	1.595 (1.283, 1.984)*	2.315 (1.698, 3.157)*	0.702 (0.14, 3.532)
Insulin Regimen: Premix	1.19 (1.022, 1.386)*	1.814 (1.463, 2.249)*	0.657 (0.14, 3.075)
Insulin Regimen: basal	Reference	Reference	Reference
log eGFR (mL/min/1.73m^2)	0.8 (0.723, 0.886)*	0.775 (0.674, 0.891)*	0.796 (0.46, 1.379)
Systolic BP (mmHg)	0.997 (0.996, 0.999)*	0.997 (0.995, 0.999)*	0.988 (0.979, 0.996)*
Diastolic BP (mmHg)	0.987 (0.985, 0.99)*	0.987 (0.984, 0.99)*	0.98 (0.966, 0.993)*
BMI (kg/m^2)	0.962 (0.959, 0.966)*	0.961 (0.956, 0.966)*	1.008 (0.986, 1.029)
HDL Cholesterol (mmol/L)	1.003 (1.002, 1.004)*	1.003 (1.002, 1.005)*	1.004 (0.995, 1.012)
LDL Cholesterol (mmol/L)	1 (0.999, 1)	1 (0.999, 1.001)	0.993 (0.987, 0.999)*
Total Cholesterol (mmol/L)	0.923 (0.904, 0.942)*	0.914 (0.888, 0.941)*	0.81 (0.7, 0.937)*
Triglycerides	0.895 (0.88, 0.91)*	0.859 (0.837, 0.882)*	0.904 (0.788, 1.038)
CVD At Baseline	0.816 (0.776, 0.859)*	0.836 (0.779, 0.898)*	2.223 (1.492, 3.314)*
Retinopathy At Baseline	0.973 (0.91, 1.04)	0.903 (0.823, 0.991)*	1.097 (0.702, 1.716)
Neuropathy At Baseline	0.917 (0.864, 0.972)*	0.93 (0.857, 1.009)	1.415 (0.996, 2.011)
Nephropathy At Baseline	0.908 (0.825, 0.999)*	0.894 (0.78, 1.025)	1.293 (0.652, 2.562)
CM: Anti-epileptic	1.207 (1.076, 1.353)*	1.309 (1.121, 1.529)*	2.918 (1.755, 4.85)*
CM: Anti-hypertensives	0.91 (0.869, 0.953)*	0.926 (0.868, 0.987)*	1.68 (1.215, 2.324)*
CM: Anti-inflammatories	1.294 (1.233, 1.357)*	1.302 (1.219, 1.391)*	2.102 (1.613, 2.738)*
CM: Blood-glucose lowering drugs	1.029 (0.955, 1.109)	0.964 (0.874, 1.063)	0.81 (0.564, 1.163)
CM: Psychoactives	1.252 (1.183, 1.324)*	1.352 (1.252, 1.459)*	3.041 (2.293, 4.034)*
CM: Sex-hormones	1.365 (1.193, 1.561)*	1.132 (0.944, 1.357)	1.808 (0.908, 3.603)
CM: Steroid cessation	0.587 (0.252, 1.367)	0.463 (0.125, 1.723)	2.832 (0.122, 65.959)

Note:
Association with Antibiotics and Anti-thyroid medications were excluded from this analysis as number of observations were low.
Blood glucose is self-monitoring blood glucose
Level 3 events were low in Human+Analogue insulin leading to wide confidence intervals
when estimating associations with insulin origin.
For continuous covariates this is the increase in hypoglycaemia rate for every standard deviate change in covariate for the first 6 weeks of the study and for categorical covariates it is the increase in hypoglycaemia rate with respect to the reference category.
In both cases adjusted for age, sex, diabetes duration and study identifier as fixed effects, and individual identifier as random effect.
* Associations where the confidence interval does not cross 1, and are therefore significant ($P < 0.05$), are highlighted in bold.

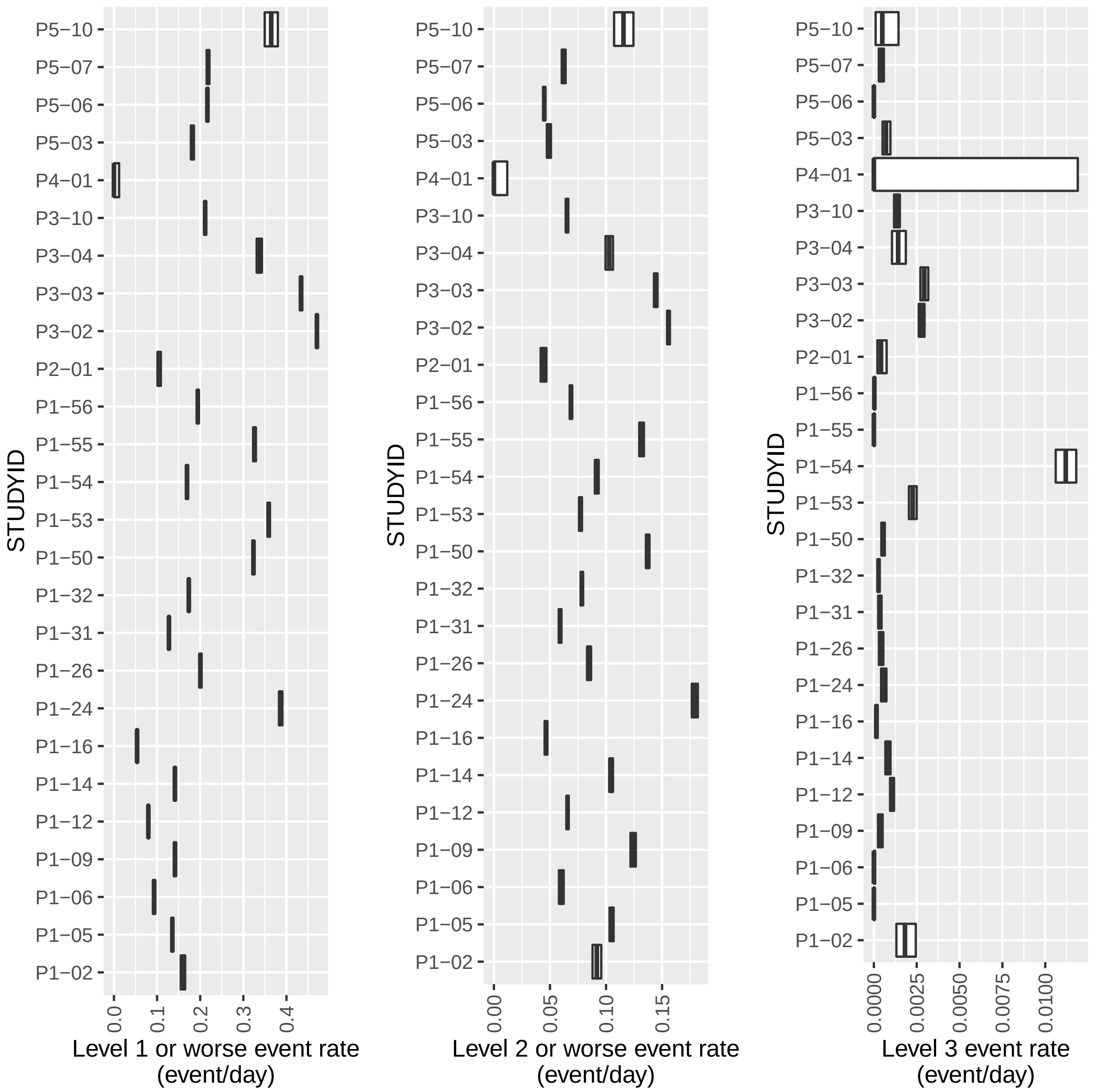

ESM Figure 1: Per-trial hypoglycaemia rates for type 1 diabetes participants with available age, sex, diabetes duration, and hypoglycaemic event data. White boxes show 95% confidence intervals, assuming a Poisson event rate, with the mean rate shown as the dividing middle black line in each box.

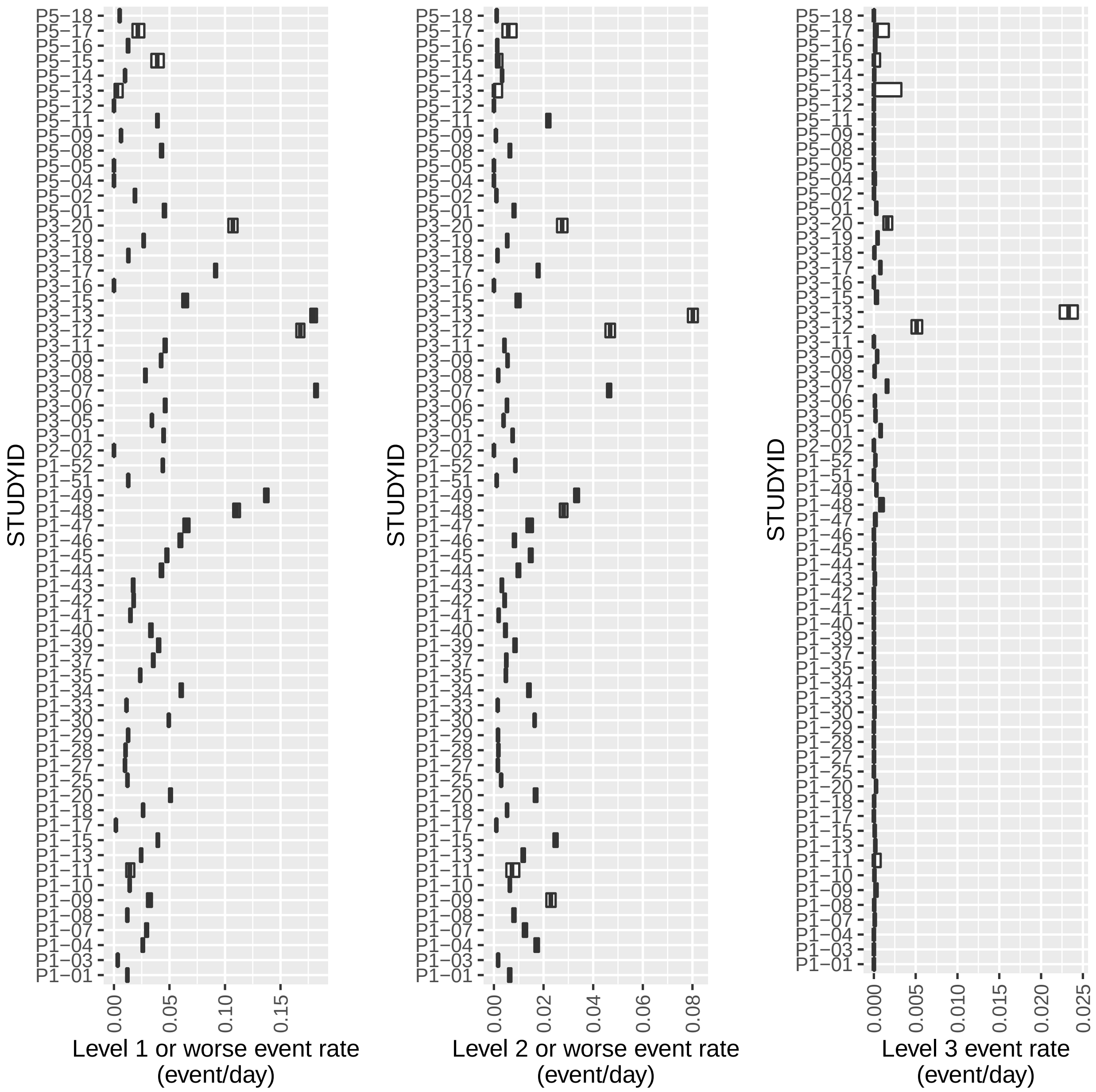

ESM Figure 2: Per-trial hypoglycaemia rates for type 2 diabetes participants with available age, sex, diabetes duration, and hypoglycaemic event data. White boxes show 95% confidence intervals, assuming a Poisson event rate, with the mean rate shown as the dividing middle black line in each box.
